# Effects of DISC1 on Alzheimer’s disease cell models assessed by iTRAQ proteomics analysis

**DOI:** 10.1042/BSR20211150

**Published:** 2022-01-11

**Authors:** Jiajie Lu, Rihong Huang, Yuecheng Peng, Haojian Wang, Zejia Feng, Yongyang Fan, Zhaorong Zeng, Yezhong Wang, Jiana Wei, Zhaotao Wang

**Affiliations:** 1 Institute of Neuroscience, Department of Neurosurgery, The Second Affiliated Hospital of Guangzhou Medical University, Guangzhou 510260, China; 2 Institute of Neuroscience, Department of Neurology, The Second Affiliated Hospital of Guangzhou Medical University, Guangzhou 510260, China

**Keywords:** Alzheimer’s disease (AD), DISC1, iTRAQ proteomic

## Abstract

Alzheimer’s disease (AD) is a form of neurodegenerative disease in the elderly with no cure at present. In a previous study, we found that the scaffold protein, disrupted in Schizophrenia 1 (DISC1) is down-regulated in the AD brains, and ectopic expression of DISC1 can delay the progression of AD by protecting synaptic plasticity and down-regulating BACE1. However, the underlying mechanisms remain not to be elucidated. In the present study, we compared the proteomes of normal and DISC1^high^ AD cells expressing the amyloid precursor protein (APP) using isobaric tag for relative and absolute quantitation (iTRAQ) and mass spectrometry (MS). The differentially expressed proteins (DEPs) were identified, and the protein–protein interaction (PPI) network was constructed to identify the interacting partners of DISC1. Based on the interaction scores, NDE1, GRM3, PTGER3 and KATNA1 were identified as functionally or physically related to DISC1, and may therefore regulate AD development. The DEPs were functionally annotated by Gene Ontology (GO) and Kyoto Encyclopedia of Genes and Genomes (KEGG) databases with the DAVID software, and the Non-supervised Orthologous Groups (eggNOG) database was used to determine their evolutionary relationships. The DEPs were significantly enriched in microtubules and mitochondria-related pathways. Gene set enrichment analysis (GSEA) was performed to identify genes and pathways that are activated when DISC1 is overexpressed. Our findings provide novel insights into the regulatory mechanisms underlying DISC1 function in AD.

## Introduction

Alzheimer’s disease (AD) is a highly prevalent neurodegenerative disease, and accounts for almost 80% of the dementia cases worldwide [1]. It commonly afflicts the elderly (>65 years), also known as the late-onset AD, and early-onset AD that affects those younger than 65 years of age is relatively rare [2]. It is characterized by the deposition of β-amyloid (Aβ) plaques and formation of neurofibrillary tangles (NFTs) of hyperphosphorylated tau protein, which impairs neuronal and synaptic functions [3–5].

Disrupted in Schizophrenia 1 (DISC1) is a multifunctional scaffold protein that is ubiquitous in the brain, and highly expressed in the temporal and para-hippocampal cortices, dentate gyrus of the hippocampus, and the white matter [6]. It interacts with multiple proteins involved in physiological processes such as neuron migration, neural progenitor cell (NPC) proliferation, neural signal transmission and synaptic functions, indicating a pathological role in neurodegeneration as well as a potential target for drug intervention [7]. Overexpression of DISC1 can reduce cognitive deficits and delay the progression of AD by protecting synaptic plasticity and down-regulating BACE1 [8,9]. However, the exact molecular mechanisms underlying the role of DISC1 in AD pathogenesis are unknown. To this end, we ectopically expressed DISC1 in the amyloid precursor protein (APP) cellular model of AD, and compared the proteomes of the control and overexpressing cells to identify the differentially expressed proteins (DEPs). Identifying proteins that interacted with DISC1 may reveal molecular mechanisms underlying DISC1 function in AD or other disorders.

## Materials and methods

### *In vitro* model of AD

The HEK293-APP cells were obtained from Institute of Neuroscience, Soochow University. The cells were transfected with the DISC1-overexpression (OE) and control lentiviruses (NC), and three replicates were used for each.

### Protein extraction and digestion

Total protein was extracted from the cells using RIPA buffer (Beyotime, China) according to the manufacturer’s protocol, and enzymatically digested with the filter-aided sample preparation (FASP) method [10]. Aliquots of lysates were mixed with 200 μl of 8 M urea in Nanosep Centrifugal Devices (PALL). The device was centrifuged at 14000×***g*** at 20°C for 20 min. All following centrifugation steps were performed applying the same conditions allowing maximal concentration. The concentrate was diluted with 200 μl of 8 M urea in 0.1 M Tris-HCl, pH 8.5 and the device was centrifuged. Proteins were reduced with 10 mM DTT for 2 h at 56°C. Subsequently, the samples were incubated in 5 mM iodoacetamide for 30 min in the dark to block reduced cysteine residues followed by centrifugation. The resulting concentrate was diluted with 200 μl of 8 M urea in 0.1 M Tris-HCl, pH 8.0 and concentrated again. This step was repeated two-times, and the concentrate was subjected to proteolytic digestion overnight at 37°C. The digests were collected by centrifugation, dried in a vacuum concentrator.

### Isobaric tag for relative and absolute quantitation labeling

The tryptic digestion peptides were labeled with 8plex isobaric tag for relative and absolute quantitation (iTRAQ) reagents according to the manufacturer’s instructions (Sciex, Foster City, CA). The dried peptides were resuspended in 200 mM triethylammonium bicarbonate (TEAB) buffer. For 50 μg peptide mixture per sample, half units of labeling reagent with respective isobaric tags from 113 to 119, which were dissolved in 140 μl isopropanol, were added into each sample tube, then vortexed for 1 min and incubated at room temperature for 2 h. The reaction was stopped with 100 µl high-performance liquid chromatography (HPLC) water for 30 min. The samples were labeled as (CON1)-113, (CON2)-114, (CON3)-115, (DISC1)-116, (DISC2)-117 and (DISC3)-118, (Mix)-119. Finally, all the samples were pooled and vacuum-dried for the next step.

### Peptides fractionation by high pH reversed-phase liquid chromatography

Agilent 1100 HPLC system was used for peptides chromatographic fractionation. The dried sample was suspended in 110 μl solvent A (10 mM ammonium formate, 5% acetonitrile aqueous solution, pH = 10) and loaded on to an analytical C18 column (Zorbax Extended-C18, 2.1*150 mm, 5 μm, Agilent). A total of 80 min separation procedure was carried out at a flow rate of 300 μl/min with the monitoring wavelength of 215 nm, the elution gradient was set as follows: keep 5% solvent B (10 mM ammonium formate, 90% acetonitrile aqueous solution, pH = 10.0) for 5 min, increase from 5 to 38% solvent B in 60 min, from 38 to 90% B in 1 min, keep 90% B for 7 min, then equilibrate the column with 5% B for 7 min. A total of 48 fractions were evenly collected within the elution gradient starting from the 6 to 65^th^ min, these 48 fractions were then pooled to generate the final 16 fractions using a nonadjacent pooling scheme (e.g., 1, 17, 49 pooled for final fraction 1; 2,18,50 pooled for final fraction 2). All the fractions were then dried in a vacuum for nano ESI-LC-MS/MS analysis.

### Nano ESI-LC-MS/MS analysis

The lyophilized peptide fractions were resuspended in ddH_2_O containing 0.1% formic acid, and 2 μl aliquots which were loaded into a nanoViper C18 (3 μm, 100 Å) trap column. The online chromatography seperation was performed on the Easy nLC 1200 system (Thermo Fisher). The trapping, desalting procedure were carried out with a volumn of 20 μl of 100% solvent A (0.1% formic acid). Then, an elution gradient of 8–38% solvent B (80% acetonitrile, 0.1% formic acid) at a flow rate of 300 nl/min (0–40 min, 5–38% B; 40–42 min, 38–100% B; 42–50 min, 100% B) in 60 min was used on an analytical column (50 μm × 15 cm C18-3 μm 100 Å). Data-dependent acquisition (DDA) mass spectrum techniques were used to acquire tandem MS data on a Thermo Fisher Q Exactive mass spectrometer (Thermo Fisher, U.S.A.) fitted with a Nano Flex ion source. Data were acquired using an ion spray voltage of 1.9 kV, and an interface heater temperature of 275°C. The mass spectrometry (MS) was operated with FULL-MS scans. For DDA, survey scans were acquired in 250 ms and up to 20 product ion scans (50 ms) were collected. Only spectra with a charge state of 2–4 were selected for fragmentation by higher energy collision energy. Dynamic exclusion was set for 25.

### Identification analysis and quantitative analysis

The MS converter software (Version 1.3 beta Sciex) was used to convert the raw MS data into an open data format for subsequent analysis. Proteins were identified using the iPEAK tool that can combine results from multiple MS/MS search engines, including MSGFDB, X!tandem and MyriMatch [11]. The local false discovery rate (FDR) was 1% after searching against the amino acid sequences with a maximum of two missed cleavages and one missed termini cleavage (semitryptic digest). The following parameters were set for database searching: trypsin digestion, carbamidomethylation (C) of cysteine, iTRAQ8plex (N-term, K) as a fixed modification, and oxidation (M) and iTRAQ8plex (Y) as variable modifications. Precursor and fragment mass tolerance were set to 20 ppm and 0.05 Da, respectively. IQuant was used to quantify the labeled peptides with isobaric tags [12] using the following parameters: Quant_peptide of all unique peptides, Quant_number of at least one unique spectrum, variance stabilization normalization, Protein_Ratio of weighted average, and *t* test for Statistical Analysis. For protein abundance ratios, fold-changes ≥ 1.2 (or ≤0.833) and *P*-values <0.05 were the thresholds for statistical significance. The data were searched in the UniProt database using *Homo sapiens* (human) taxonomy (https://www.uniprot.org/proteomes/UP000005640).

### Protein–protein interaction network construction

The STRING (version: 11.0b, https://string-db.org) search tool [13] was used to identify the key interacting proteins upstream and downstream of DISC1. Briefly, DEPs are imported into the database, and protein pairs with a total score > 0.4 were used to construct the protein–protein interaction (PPI) network that was further visualized by Cytoscape.

### Gene Ontology, Kyoto Encyclopedia of Genes and Genomes and non-supervised Orthologous Groups analyses

Gene Ontology (GO), Kyoto Encyclopedia of Genes and Genomes (KEGG) and Non-supervised Orthologous Groups (eggNOG) analyses were performed using Bubble and Barplot Color Group tools in Hiplot basic model (https://hiplot.com.cn).

### Enrichment statistical analysis of DEPs

The target genes and their associated functions were identified from GO database based on the biological process (BP), molecular function (MF) and cellular component (CC) categories. The various pathways involved in the DEPs were identified using the KEGG database. DAVID database tool (https://david.ncifcrf.gov/) [14,15] was used to identify the significantly enriched functions and pathways with *P*<0.05 as the threshold. The evolutionary genealogy of genes encoding for the DEPS was analyzed using the eggNOG [16] database with *P*<0.05 as statistically significant.

### Gene set enrichment analysis

**Figure 1:**
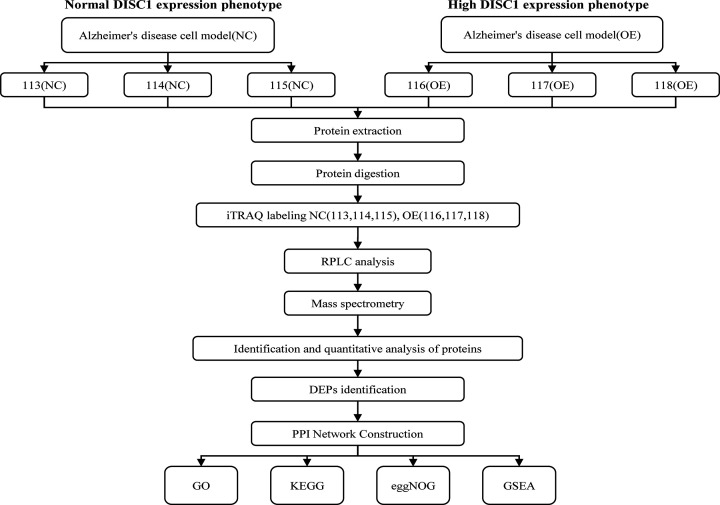
Schematic representation of the experimental design

Gene set enrichment analysis (GSEA) was performed to screen for the gene sets and pathways enriched in the DISC1^high^ AD cells to identify genes associated with disease progression [17,18]. R clusterProfiler package [19] was used for GSEA on the basis of BP, MF, CC and KEGG categories. For each analysis, gene set permutations were implemented 5000 times. Gene sets with a FDR < 0.05 and adjusted *P*-value <0.05 were considered significantly enriched. The workflow of the study is outlined in Figure 1.

## Results

### DEPs screening and PPI network construction

**Figure 2:**
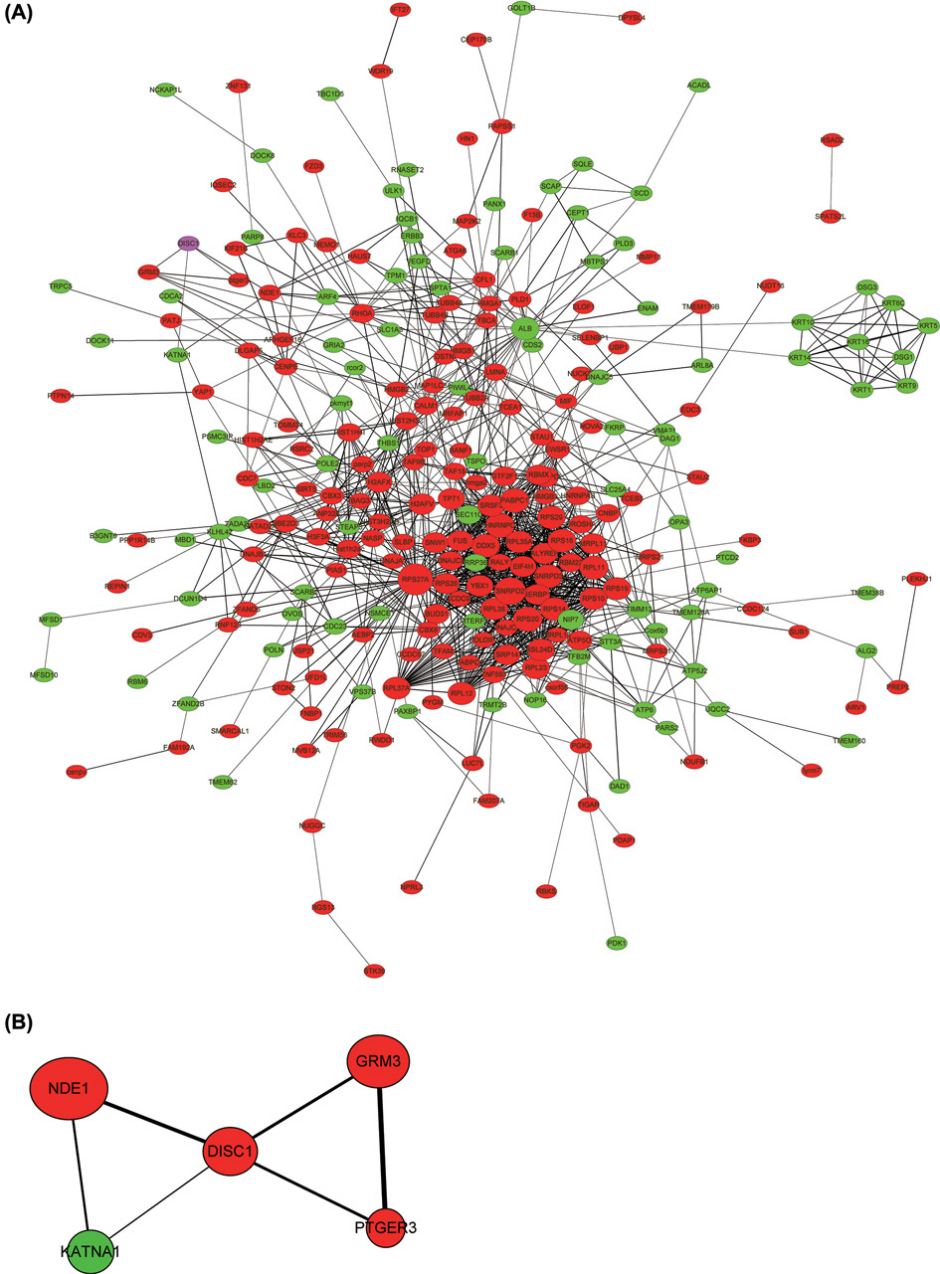
PPI network of DEPs (**A**) The PPI network of DEPs was constructed by STRING and Cytoscape. Red nodes indicate up-regulated genes, green nodes indicate down-regulated genes. The node size correlates to the degree of connectivity and line thickness with interaction score. DISC1 is marked in purple in the upper left corner. (**B**) Proteins with physical or functional association with DISC1.

Firstly, we detected the DISC1 expression level after overexpression of DISC1 in HEK-APP cells by Western blot. And we found DISC1 is up-regulated in HEK-APP cells compared with the control group (Supplementary Figure S1). Besides, according the results from our proteomics, DISC1 is overexpressed ∼2.18-folds than the control group. Then, a total of 351 DEPs (Supplementary Table S1) were identified in the DISC1^high^ AD cells relative to the control cells, and the top 20 up-regulated and down-regulated proteins are listed in Tables 1 and 2, respectively. The PPI network of the DEPs was then constructed using the STRING database to identify interacting partners (Figure 2A). As shown in Figure 2B, DISC1 directly or indirectly interacts with NDE1, GRM3, PTGER3 and KATNA1 at a physical and/or functional level.

**Table 1:** Top 20 up-regulated DEPs in AD cells overexpressing DISC1

Protein ID	Gene symbol	Description	Mean ratio
MRPL14	*PFN3*	Profilin-3	2.663172018
P62273	*RPS29*	40S ribosomal protein S29	2.481425328
Q9NRI5	*DISC1*	Disrupted in schizophrenia 1 protein	2.175152568
Q9UK76	*JPT1*	Jupiter microtubule-associated homolog 1	2.129304373
D6RIA3	*C4orf54*	Uncharacterized protein C4orf54	1.976621657
Q6P1L8	*MRPL14*	39S ribosomal protein L14, mitochondrial	1.946385443
Q71DI3	*HIST2H3A*	Histone H3.2	1.807106261
P05160	*F13B*	Coagulation factor XIII B chain	1.656737482
Q93077	*HIST1H2AC*	Histone H2A type 1-C	1.604556122
Q8WXG1	*RSAD2*	Radical S-adenosyl methionine domain-containing protein 2	1.593975355
P82921	*MRPS21*	28S ribosomal protein S21, mitochondrial	1.577483389
O75037	*KIF21B*	Kinesin-like protein KIF21B	1.570883653
Q9UK80	*USP21*	Ubiquitin carboxyl-terminal hydrolase 21	1.566709143
P52926	*HMGA2*	High mobility group protein HMGI-C	1.56653705
Q9BY77	*POLDIP3*	Polymerase δ-interacting protein 3	1.562567582
Q9Y6C2	*EMILIN1*	EMILIN-1	1.562523246
O00488	*ZNF593*	Zinc finger protein 593	1.561342328
Q9H446	*RWDD1*	RWD domain-containing protein 1	1.551293724
Q9NW61	*PLEKHJ1*	Pleckstrin homology domain-containing family J member 1	1.544695272
Q13442	*PDAP1*	28 kDa heat- and acid-stable phosphoprotein	1.542954482

**Table 2:** Top 20 down-regulated DEPs in AD cells overexpressing DISC1

Protein ID	Gene symbol	Description	Mean ratio
O95674	*CDS2*	Phosphatidate cytidylyltransferase 2	0.490415498
Q9H8M5	*CNNM2*	Metal transporter CNNM2	0.537252179
P78369	*CLDN10*	Claudin-10	0.553699125
Q9UL62	*TRPC5*	Short transient receptor potential channel 5	0.560397651
Q92858	*ATOH1*	Protein atonal homolog 1	0.561748688
P48668	*KRT6C*	Keratin, type II cytoskeletal 6C	0.56390028
P51786	*ZNF157*	Zinc finger protein 157	0.586087661
P13647	*KRT5*	Keratin, type II cytoskeletal 5	0.611197075
Q5HY64	*FAM47C*	Putative protein FAM47C	0.625502861
P08779	*KRT16*	Keratin, type I cytoskeletal 16	0.633121401
P21860	*ERBB3*	Receptor tyrosine-protein kinase erbB-3	0.643736939
P04264	*KRT1*	Keratin, type II cytoskeletal 1	0.656660409
P78332	*RBM6*	RNA-binding protein 6	0.656667186
Q96EU6	*RRP36*	Ribosomal RNA processing protein 36 homolog	0.680711277
Q9NX00	*TMEM160*	Transmembrane protein 160	0.685147795
Q92564	*DCUN1D4*	DCN1-like protein 4	0.691796699
Q9P003	*CNIH4*	Protein cornichon homolog 4	0.696978494
P80365	*HSD11B2*	Corticosteroid 11-beta-dehydrogenase isozyme 2	0.700629987
P28330	*ACADL*	Long-chain specific acyl-CoA dehydrogenase, mitochondrial	0.707497167
Q02413	*DSG1*	Desmoglein-1	0.707750707

### Enrichment analysis of DEPs

**Figure 3:**
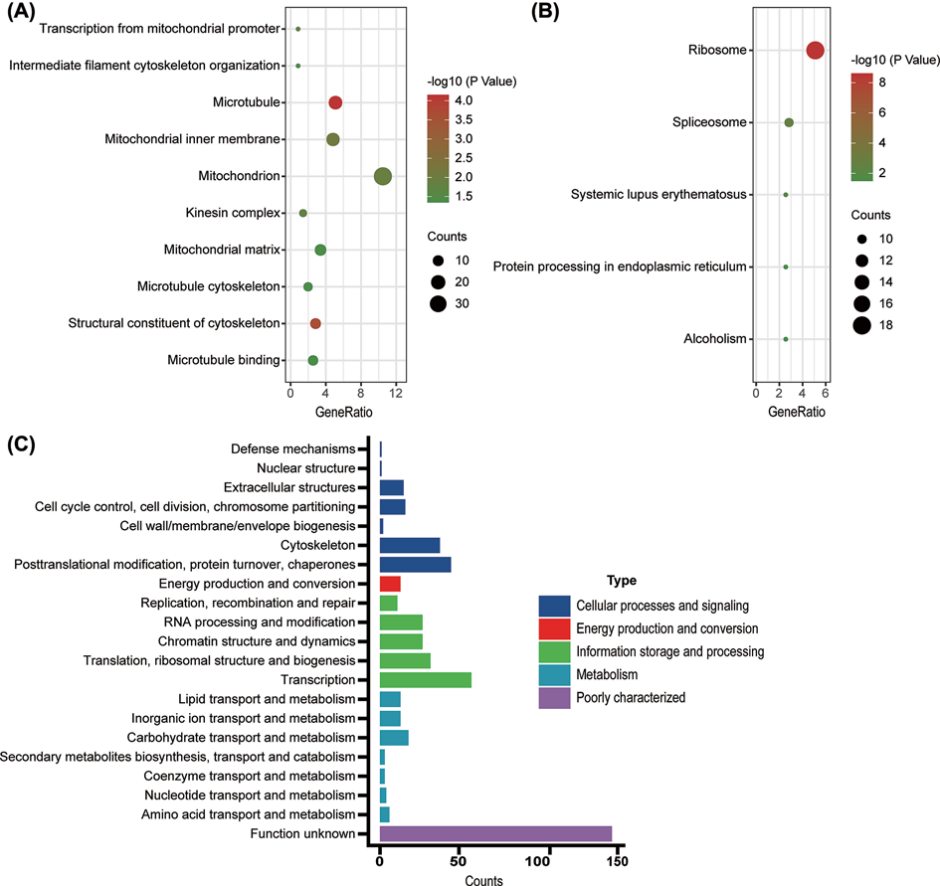
The enrichment analysis of DEPs Functional enrichment analysis of GO (**A**). Functional enrichment analysis of KEGG (**B**). Functional enrichment analysis of eggNOG (**C**).

The DEPs were functionally annotated with GO, KEGG and eggNOG enrichment analyses. As shown in Figure 3A, the DEPs were significantly enriched in transcription, mitochondrial promoter, intermediate filament cytoskeleton organization, microtubule, mitochondrial inner membrane and mitochondrion etc. Furthermore, KEGG pathway analysis further showed that the ribosome, spliceosome, systemic lupus erythematosus, protein processing in the endoplasmic reticulum and Alcoholism pathways were significantly associated with the DEPs (Figure 3B) In additon, eggNOG enrichment analyses showed that posttranslational modification and translation were closely related to the DEPs (Figure 3C). The results are summarized in Table 3.

**Table 3:** GO and KEGG pathway analyses of hub genes

Category	Term	Counts	GeneRatio	*P*-value
GOTERM_BP_DIRECT	GO:0006390∼transcription from mitochondrial promoter	3	0.849858	0.014464
GOTERM_BP_DIRECT	GO:0045104∼intermediate filament cytoskeleton organization	3	0.849858	0.031722
GOTERM_CC_DIRECT	GO:0005874∼microtubule	18	5.09915	7.06E-05
GOTERM_CC_DIRECT	GO:0005743∼mitochondrial inner membrane	17	4.815864	0.008035
GOTERM_CC_DIRECT	GO:0005739∼mitochondrion	37	10.48159	0.013376
GOTERM_CC_DIRECT	GO:0005871∼kinesin complex	5	1.416431	0.016361
GOTERM_CC_DIRECT	GO:0005759∼mitochondrial matrix	12	3.399433	0.041063
GOTERM_CC_DIRECT	GO:0015630∼microtubule cytoskeleton	7	1.983003	0.041571
GOTERM_MF_DIRECT	GO:0005200∼structural constituent of cytoskeleton	10	2.832861	2.45E-04
GOTERM_MF_DIRECT	GO:0008017∼microtubule binding	9	2.549575	0.045076
KEGG_PATHWAY	hsa03010:Ribosome	18	5.09915	2.44E-09
KEGG_PATHWAY	hsa03040:Spliceosome	10	2.832861	0.001809
KEGG_PATHWAY	hsa05322:Systemic lupus erythematosus	9	2.549575	0.006885
KEGG_PATHWAY	hsa04141:Protein processing in endoplasmic reticulum	9	2.549575	0.025116
KEGG_PATHWAY	hsa05034:Alcoholism	9	2.549575	0.031921

### Identification of AD-related genes associated with DISC1

**Figure 4:**
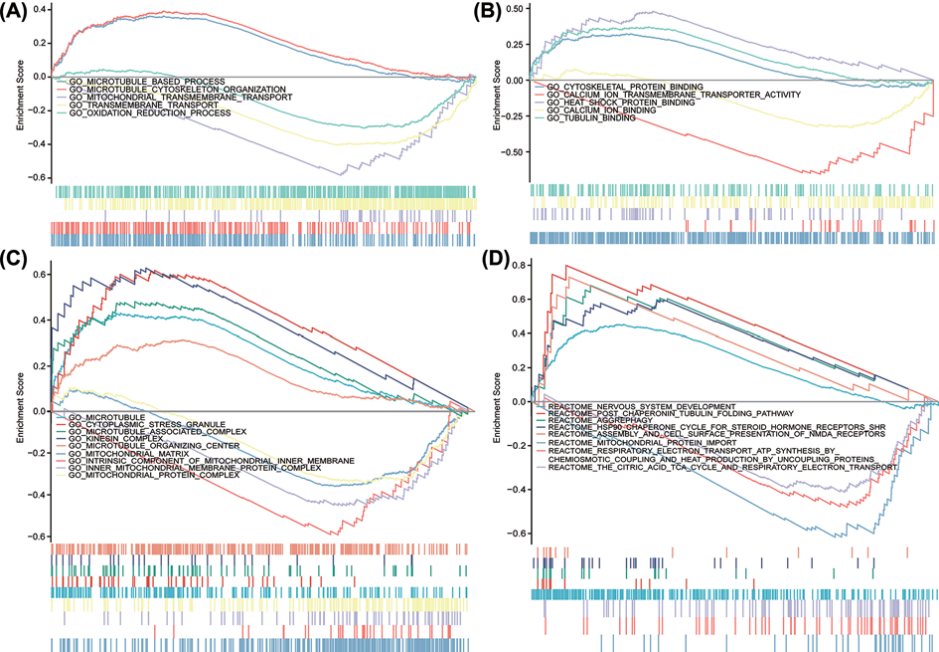
Enrichment plots from GSEA in DISC1^high^ phenotype GSEA results showing DEPs in BP (**A**), MF (**B**), CC (**C**) and REACTOME (**D**). All results of GSEA were based on NES, adjusted *P*-value and FDR value.

GSEA was performed to identify the AD-related gene sets in the DISC1^high^ AD cells. Significant differences were observed in the enriched GO terms and KEGG pathways of the DISC1^low^ and DISC1^high^ datasets. Microtubule-based process, microtubule cytoskeleton organization, mitochondrial transmembrane transport, transmembrane transport and oxidation–reduction were the significantly enriched BP categories in DISC1^high^ cells (Figure 4A), and the top five MF categories were cytoskeletal protein binding, calcium ion transmembrane transporter activity, heat shock protein (Hsp) binding, calcium ion binding and tubulin binding (Figure 4B). In addition, the DISC1^high^ phenotype showed significant enrichment of microtubule, cytoplasmic stress granule, microtubule-associated complex, kinesin complex and microtubule organizing center, along with a significant negative correlation with mitochondrial matrix, mitochondrial inner membrane component, inner mitochondrial membrane protein complex and mitochondrial protein complex (Figure 4C). Five REACTOME categories including nervous system development, post-chaperonin tubulin folding pathway, aggrephagy, Hsp90 chaperone cycle for steroid hormone receptors SHR, and the assembly and cell surface presentation of NMDA receptor were significantly enriched in the DISC1^high^ phenotype. While, mitochondrial protein import, respiratory electron transport ATP synthesis by chemiosmotic coupling and heat production by uncoupling proteins, and the citric acid TCA cycle and respiratory electron transport were negatively associated with this phenotype (Figure 4D). The results are summarized in Table 4.

**Table 4:** Gene sets enriched in high DISC1 expression phenotype

Gene set name	NES	*P*.adjust	FDR
BP			
GO_MICROTUBULE_BASED_PROCESS	1.619	0.032	0.03
GO_MICROTUBULE_CYTOSKELETON_ORGANIZATION	1.695	0.032	0.03
GO_MITOCHONDRIAL_TRANSMEMBRANE_TRANSPORT	−2.064	0.032	0.03
GO_TRANSMEMBRANE_TRANSPORT	−2.015	0.035	0.032
GO_OXIDATION_REDUCTION_PROCESS	−1.524	0.035	0.032
MF			
GO_CYTOSKELETAL_PROTEIN_BINDING	1.472	0.011	0.009
GO_CALCIUM_ION_TRANSMEMBRANE_TRANSPORTER _ACTIVITY	−2.085	0.011	0.009
GO_HEAT_SHOCK_PROTEIN_BINDING	1.748	0.031	0.025
GO_CALCIUM_ION_BINDING	−1.481	0.038	0.031
GO_TUBULIN_BINDING	1.533	0.047	0.038
CC			
GO_MICROTUBULE	1.813	0.011	0.009
GO_CYTOPLASMIC_STRESS_GRANULE	2.059	0.011	0.009
GO_MICROTUBULE_ASSOCIATED_COMPLEX	1.783	0.015	0.012
GO_KINESIN_COMPLEX	1.928	0.015	0.012
GO_MICROTUBULE_ORGANIZING_CENTER	1.401	0.049	0.04
GO_MITOCHONDRIAL_MATRIX	−1.754	0.014	0.011
GO_INTRINSIC_COMPONENT_OF_MITOCHONDRIAL_ INNER_MEMBRANE	−1.971	0.018	0.015
−GO_INNER_MITOCHONDRIAL_MEMBRANE_PROTEIN _COMPLEX	−1.725	0.018	0.015
GO_MITOCHONDRIAL_PROTEIN_COMPLEX	−1.548	0.026	0.021
REACTOME			
REACTOME_NERVOUS_SYSTEM_DEVELOPMENT	2.004	0.021	0.019
REACTOME_POST_CHAPERONIN_TUBULIN_FOLDING _PATHWAY	1.947	0.021	0.019
REACTOME_AGGREPHAGY	1.946	0.037	0.032
REACTOME_HSP90_CHAPERONE_CYCLE_FOR_ STEROID_HORMONE_RECEPTORS_SHR	1.89	0.045	0.04
REACTOME_ASSEMBLY_AND_CELL_SURFACE_ PRESENTATION_OF_NMDA_RECEPTORS	1.885	0.045	0.04
REACTOME_MITOCHONDRIAL_PROTEIN_IMPORT	−2.097	0.021	0.019
REACTOME_RESPIRATORY_ELECTRON_TRANSPORT_ATP_ SYNTHESIS_BY_CHEMIOSMOTIC_COUPLING_AND_ HEAT_PRODUCTION_BY_UNCOUPLING_PROTEINS	−1.782	0.045	0.04
REACTOME_THE_CITRIC_ACID_TCA_CYCLE_ AND_RESPIRATORY_ELECTRON_TRANSPORT	−1.676	0.048	0.042

Abbreviations: NES, normalized enrichment score; *P*.adjust, adjusted *P*-value.

## Discussion

AD is the main cause of dementia and is a considerable socioeconomic burden worldwide [20]. The histological hallmarks of AD are extracellular accumulation of senile Aβ plaques, formation of intracellular NFTs, and glial cell-mediated inflammation [21,22]. However, the molecular mechanisms driving the pathophysiology of AD have not completely elucidated. We previously showed that DISC1 slowed AD development by protecting synaptic plasticity and down-regulating BACE1 [8,9]. In the present study, we compared the proteomes of DISC1^low^ and DISC1^high^ AD cells to identify the proteins differentially expressed in response to DISC1 up-regulation. Through bioinformatics analysis, we identified key DSIC1-interacting proteins and pathways, mostly related to microtubules and mitochondria, that likely play important roles in the progression of AD.

Several studies have implicated microtubules in neurodegenerative diseases. Zempel et al. showed that the loss of microtubules is a risk factor in the progression of AD [23]. In our study, the DEPs and the gene sets correlated to DISC1 were significantly enriched in the microtubule and cytoskeleton-related functions and pathways. Furthermore, NDE1 and KATNA1, the proteins with high interaction score with DISC1, are involved in the microtubule and microtubule-binding pathways. Studies show that NDE1 is aberrantly expressed in neurological and psychiatric disorders [24,25], and binds to DISC1 to mediate the latter’s role in neurogenesis and neural development [7,26]. In addition, KATNA1 regulates neuronal progenitor proliferation during embryonic development and adult neurogenesis [27]. Thus, DISC1 may directly or indirectly regulate NDE1 and KATNA1, and the downstream microtubule-related pathways to slow down the progression of AD.

The mitochondrial cascade hypothesis for AD was first proposed in 2004, and considers mitochondrial dysfunction as the prerequisite of events leading to AD [3]. Several studies have subsequently shown that mitochondrial dysfunction and oxidative stress play a central role in AD pathogenesis [28–31]. In our previous study, we reported that DISC1 functions as a mitophagy receptor that can clear the dysfunctional mitochondria [8]. In this study, we found that DEPs were significantly enriched in mitochondria-related functions and pathways. In addition, the negative NES values of these pathways in GSEA indicated that mitochondria-related functions are inhibited when DISC1 is highly expressed. Furthermore, cytoplasmic stress granule was an enriched CC category in GSEA, which is indicative of oxidative stress in the cytoplasm of AD cells. Therefore, DISC1 slows the progression of AD maybe by clearing the damaged mitochondria.

Pathways related to the kinesin complex, intracellular trafficking, vesicular transport and calcium influx were also enriched in the DEPs correlated to DISC1. Stokin et al. found that axonal lesions and defective microtubule-dependent transport are early pathological signs of AD, and that reduction in kinesin-I levels can increase Aβ generation and intraneuronal accumulation [32]. Besides, Murphy et al. demonstrated that the regulation of mitochondrial dynamics by DISC1, which is DISC1 robustly associates with mitochondrial trafficking on microtubule complexes, through multiple protein interaction, including DISC1, NDE1, kinesin complex etc., is a putative risk factor for major mental illness [33]. Furthermore, kinesin is also involved in the transport of mitochondria along axons, which is disrupted in neurodegenerative diseases [34–37]. Zempel et al. showed that calcium influx is also a significant factor in the pathogenesis of AD [23], which was validated by the enrichment of intracellular trafficking, secretion and vesicular transport by eggNOG analysis in our study. The pathways related to Hsp, aggrephagy and NMDAR were also enriched receptors among the DISC1-interacting proteins. Studies show that Hsp70 and Hsp90 can promote the clearance of Aβ plaques and delay the progression of AD [38–40]. Aggrephagy refers to the selective clearance of protein aggregates by autophagy [41]. The Aβ and tau aggregates are not effectively cleared due to dysfunctional aggrephagy, resulting in the formation of aggresomes that accelerate AD progression. Little is known regarding the mechanisms underlying aggrephagy, although there is evidence indicating that DISC1 can enhance this process [42]. Wang et al. found that the activation of synaptic NMDARs initiates plasticity and promotes neuronal survival, thereby slowing the progression of AD [43].

The other interacting partners of DISC1 identified by the PPI network were GRM3 and PTGER3 (interaction scores 0.630 and 0.627, respectively), which may also regulate DISC1 during AD progression. The GRM3 gene encodes mGluR3, which regulates neuronal and glial functions, as well as neuronal excitability and synaptic transmission [44]. Caraci et al. found that mGluR3 down-regulation and/or inactivation is correlated to impaired cognition in AD. The protective effect of mGluR3 against Aβ toxicity has also been observed in various animal models of AD, suggesting that age-related reduction in mGluR3 may contribute to the increased risk of AD [45]. Furthermore, Jin et al. found that postsynaptic mGluR3 strengthens working memory networks, and its inactivation erodes cognitive abilities [46]. PTGER3 is one of the four receptors of prostaglandin E2 (PGE2), a byproduct of arachidonic acid metabolism in the cyclooxygenase pathway, and is ubiquitously expressed in the brain [47,48]. Studies show that activation of PTGER3 can reduce or suppress cyclic adenosine monophosphate (cAMP) formation and may counteract its up-regulation via PTGER2, which has been linked to the anti-inflammatory effects of PGs. Altered PTGER3 expression in the microglia leads to acute or chronic microglial activation in brain diseases like AD [49].

In conclusion, DISC1 exerts a neuroprotective role during AD progression by interacting with NDE1, KATNA1, GRM3 and PTGER3, and regulating pathways related to microtubule function, mitochondrial dynamics, kinesin complex, calcium ion influx, Hsps, aggrephagy and NMDAR. Our findings will have to be verified by experimental studies. Nevertheless, the present study provides novel insights into the mechanisms driving AD progression, along with potential therapeutic targets that can revolutionize the individualized treatment of AD patients.

## Supplementary Material

Supplementary Figure S1 and Table S1

## Data Availability

The datasets used and/or analyzed during the current study are available from the corresponding authors on reasonable request.

## Abbreviations

AD: Alzheimer’s disease
APP: amyloid precursor protein
Aβ: β-amyloid
BP: biological process
CC: cellular component
DDA: data-dependent acquisition
DEP: differentially expressed protein
DISC1: disrupted in schizophrenia 1
eggNOG: non-supervised Orthologous Groups
FDR: false discovery rate
GO: Gene Ontology
GSEA: gene set enrichment analysis
HPLC: high-performance liquid chromatography
Hsp: heat shock protein
iTRAQ: isobaric tag for relative and absolute quantitation
KEGG: Kyoto Encyclopedia of Genes and Genomes
MF: molecular function
MS: mass spectrometry
NFT: neurofibrillary tangle
PPI: protein–protein interaction
