# Supplementary material for: Effects of DISC1 on Alzheimer’s disease cell models assessed by iTRAQ proteomics analysis
Source: Biosci Rep. 2022 Jan 11;42(1):BSR20211150. doi: 10.1042/BSR20211150 (PMC8753346; doi:10.1042/BSR20211150)
Supplement: Supplementary Figure S1 and Table S1 [file BSR-2021-1150_supp.pdf]

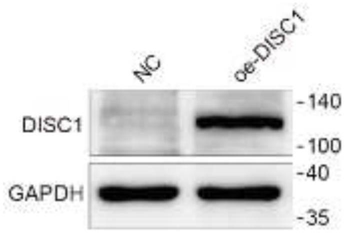


**Supplementary Figure. S1**

Western blot analysis of levels of DISC1protein in HEK293-APP cells which were transfected with DISC1-overexpression (OE) or control lentiviruses (vector).

**Supplementary Table. S1 351 DEPs in model cells of AD over-expressing DISC1.**

| Protein ID | Gene symbol | Description | Mean ratio |
|---|---|---|---|
| P60673 | PFN3 | Profilin-3 | 2.663172018 |
| P62273 | RPS29 | 40S ribosomal protein S29 | 2.481425328 |
| Q9NRI5 | DISC1 | Disrupted in schizophrenia 1 protein | 2.175152568 |
| Q9UK76 | JPT1 | Jupiter microtubule associated homolog 1 | 2.129304373 |
| D6RIA3 | C4orf54 | Uncharacterized protein C4orf54 | 1.976621657 |
| Q6P1L8 | MRPL14 | 39S ribosomal protein L14, mitochondrial | 1.946385443 |
| Q71DI3 | HIST2H3A | Histone H3.2 | 1.807106261 |
| P05160 | F13B | Coagulation factor XIII B chain | 1.656737482 |
| Q93077 | HIST1H2AC | Histone H2A type 1-C | 1.604556122 |
| Q8WXG1 | RSAD2 | Radical S-adenosyl methionine domain-containing protein 2 | 1.593975355 |
| P82921 | MRPS21 | 28S ribosomal protein S21, mitochondrial | 1.577483389 |
| O75037 | KIF21B | Kinesin-like protein KIF21B | 1.570883653 |
| Q9UK80 | USP21 | Ubiquitin carboxyl-terminal hydrolase 21 | 1.566709143 |
| P52926 | HMGA2 | High mobility group protein HMGI-C | 1.56653705 |
| Q9BY77 | POLDIP3 | Polymerase delta-interacting protein 3 | 1.562567582 |
| Q9Y6C2 | EMILIN1 | EMILIN-1 | 1.562523246 |
| O00488 | ZNF593 | Zinc finger protein 593 | 1.561342328 |
| Q9H446 | RWDD1 | RWD domain-containing protein 1 | 1.551293724 |
| Q9NW61 | PLEKHJ1 | Pleckstrin homology domain-containing family J member 1 | 1.544695272 |
| Q13442 | PDAP1 | 28 kDa heat- and acid-stable phosphoprotein | 1.542954482 |
| O95793 | STAU1 | Double-stranded RNA-binding protein Staufen homolog 1 | 1.52148379 |
| O14921 | RGS13 | Regulator of G-protein signaling 13 | 1.518755395 |
| P35637 | FUS | RNA-binding protein FUS | 1.51176635 |
| Q8WXE9 | STON2 | Stonin-2 | 1.510145096 |
| P04908 | HIST1H2AB | Histone H2A type 1-B/E | 1.499748278 |
| Q9NZC9 | SMARCAL1 | SWI/SNF-related matrix-associated actin-dependent regulator of chromatin subfamily A-like protein 1 | 1.484634212 |
| P60866 | RPS20 | 40S ribosomal protein S20 | 1.471888769 |
| P62913 | RPL11 | 60S ribosomal protein L11 | 1.469239167 |
| O00311 | CDC7 | Cell division cycle 7-related protein kinase | 1.459651794 |
| Q99871 | HAUS7 | HAUS augmin-like complex subunit 7 | 1.452152209 |
| Q5VWW1 | C1QL3 | Complement C1q-like protein 3 | 1.429847408 |
| P84103 | SRSF3 | Serine/arginine-rich splicing factor 3 | 1.425031204 |
| P09429 | HMGB1 | High mobility group protein B1 | 1.421077504 |
| P41223 | BUD31 | Protein BUD31 homolog | 1.417104628 |
| Q9NZP6 | NPAP1 | Nuclear pore-associated protein 1 | 1.405854464 |
| Q02224 | CENPE | Centromere-associated protein E | 1.391536488 |
| Q5XPI4 | RNF123 | E3 ubiquitin-protein ligase RNF123 | 1.38593246 |
| Q9H492 | MAP1LC3A | Microtubule-associated proteins 1A/1B light | 1.384994248 |

| | | | |
|---|---|---|---|
| | | chain 3A | |
| Q9UKM9 | RALY | RNA-binding protein Raly | 1.379526179 |
| Q96DE0 | NUDT16 | U8 snoRNA-decapping enzyme | 1.378987515 |
| Q6FIF0 | ZFAND6 | AN1-type zinc finger protein 6 | 1.377644089 |
| Q71UI9 | H2AFV | Histone H2A.V | 1.367391837 |
| P62263 | RPS14 | 40S ribosomal protein S14 | 1.364455781 |
| Q9UNW9 | NOVA2 | RNA-binding protein Nova-2 | 1.361265134 |
| P62633 | CNBP | Cellular nucleic acid-binding protein | 1.358100889 |
| P38159 | RBMX | RNA-binding motif protein, X chromosome | 1.358088598 |
| P84243 | H3F3A | Histone H3.3 | 1.357848195 |
| P25685 | DNAJB1 | DnaJ homolog subfamily B member 1 | 1.357253446 |
| P60002 | ELOF1 | Transcription elongation factor 1 homolog | 1.357118803 |
| P62316 | SNRPD2 | Small nuclear ribonucleoprotein Sm D2 | 1.350704621 |
| Q9NUL3 | STAU2 | Double-stranded RNA-binding protein Staufen homolog 2 | 1.350569347 |
| P45452 | MMP13 | Collagenase 3 | 1.348205049 |
| P26583 | HMGB2 | High mobility group protein B2 | 1.347960919 |
| Q14493 | SLBP | Histone RNA hairpin-binding protein | 1.339336978 |
| P62805 | HIST1H4A | Histone H4 | 1.338675082 |
| P60981 | DSTN | Destrin | 1.338360807 |
| Q14832 | GRM3 | Metabotropic glutamate receptor 3 | 1.337731441 |
| Q9NW64 | RBM22 | Pre-mRNA-splicing factor RBM22 | 1.33489686 |
| P30050 | RPL12 | 60S ribosomal protein L12 | 1.327390437 |
| Q86TN4 | TRPT1 | tRNA 2'-phosphotransferase 1 | 1.32606524 |
| Q9NXW9 | ALKBH4 | Alpha-ketoglutarate-dependent dioxygenase alkB homolog 4 | 1.325937978 |
| Q9NPG1 | FZD3 | Frizzled-3 | 1.322624257 |
| O75925 | PIAS1 | E3 SUMO-protein ligase PIAS1 | 1.321503766 |
| Q01844 | EWSR1 | RNA-binding protein EWS | 1.319601261 |
| Q53QV2 | LBH | Protein LBH | 1.318861536 |
| O75531 | BANF1 | Barrier-to-autointegration factor | 1.314593528 |
| Q00688 | FKBP3 | Peptidyl-prolyl cis-trans isomerase FKBP3 | 1.313956247 |
| Q6NXN4 | DPY19L2P1 | Putative C-mannosyltransferase DPY19L2P1 | 1.310855929 |
| Q9H8W4 | PLEKHF2 | Pleckstrin homology domain-containing family F member 2 | 1.309976401 |
| P61077 | UBE2D3 | Ubiquitin-conjugating enzyme E2 D3 | 1.305305759 |
| Q13393 | PLD1 | Phospholipase D1 | 1.304721135 |
| O14531 | DPYSL4 | Dihydropyrimidinase-related protein 4 | 1.304654158 |
| Q9HC52 | CBX8 | Chromobox protein homolog 8 | 1.304094296 |
| Q8NH81 | OR10G6 | Olfactory receptor 10G6 | 1.30345953 |
| Q9HBM6 | TAF9B | Transcription initiation factor TFIID subunit 9B | 1.302202026 |
| Q9NZP5 | OR5AC2 | Olfactory receptor 5AC2 | 1.298908529 |
| Q96EK6 | GNPNAT1 | Glucosamine 6-phosphate N-acetyltransferase | 1.297054425 |
| P17096 | HMGA1 | High mobility group protein HMG-I/HMG-Y | 1.290807567 |
| Q9NRR4 | DROSHA | Ribonuclease 3 | 1.290265936 |
| O15061 | SYNM | Synemin | 1.289195829 |
| Q9NXR1 | NDE1 | Nuclear distribution protein nudE homolog 1 | 1.288709326 |

| | | | |
|---|---|---|---|
| P11940 | PABPC1 | Polyadenylate-binding protein 1 | 1.285071017 |
| Q7Z7K6 | CENPV | Centromere protein V | 1.283916595 |
| Q68CJ6 | NUGGC | Nuclear GTPase SLIP-GC | 1.283641361 |
| P18077 | RPL35A | 60S ribosomal protein L35a | 1.283026404 |
| Q96CT7 | CCDC124 | Coiled-coil domain-containing protein 124 | 1.281811492 |
| P62318 | SNRPD3 | Small nuclear ribonucleoprotein Sm D3 | 1.281563466 |
| Q96F86 | EDC3 | Enhancer of mRNA-decapping protein 3 | 1.280314048 |
| Q5VV41 | ARHGEF16 | Rho guanine nucleotide exchange factor 16 | 1.279495682 |
| Q9UN81 | L1RE1 | LINE-1 retrotransposable element ORF1 protein | 1.279078071 |
| O75937 | DNAJC8 | DnaJ homolog subfamily C member 8 | 1.275320043 |
| P61513 | RPL37A | 60S ribosomal protein L37a | 1.27469655 |
| Q96N11 | C7orf26 | Uncharacterized protein C7orf26 | 1.272927269 |
| P23193 | TCEA1 | Transcription elongation factor A protein 1 | 1.266354628 |
| Q9UGN5 | PARP2 | Poly [ADP-ribose] polymerase 2 | 1.263690485 |
| P68371 | TUBB4B | Tubulin beta-4B chain | 1.263359478 |
| Q99729 | HNRNPAB | Heterogeneous nuclear ribonucleoprotein A/B | 1.261724378 |
| Q4J6C6 | PREPL | Prolyl endopeptidase-like | 1.261697724 |
| Q96EY5 | MVB12A | Multivesicular body subunit 12A | 1.261182618 |
| Q9BRZ2 | TRIM56 | E3 ubiquitin-protein ligase TRIM56 | 1.260905818 |
| Q8IYE0 | CCDC146 | Coiled-coil domain-containing protein 146 | 1.260509627 |
| Q9H2C2 | ARV1 | Protein ARV1 | 1.260370968 |
| Q562E7 | WDR81 | WD repeat-containing protein 81 | 1.260227986 |
| Q9H477 | RBKS | Ribokinase | 1.260169904 |
| O15347 | HMGB3 | High mobility group protein B3 | 1.258228304 |
| Q9BWE0 | REPIN1 | Replication initiator 1 | 1.257249586 |
| P46783 | RPS10 | 40S ribosomal protein S10 | 1.256715091 |
| Q12980 | NPRL3 | GATOR complex protein NPRL3 | 1.255275227 |
| Q9Y3S2 | ZNF330 | Zinc finger protein 330 | 1.254775718 |
| Q9NZI7 | UBP1 | Upstream-binding protein 1 | 1.254692752 |
| Q9NXA8 | SIRT5 | NAD-dependent protein deacylase sirtuin-5, mitochondrial | 1.253462074 |
| P07910 | HNRNPC | Heterogeneous nuclear ribonucleoproteins C1/C2 | 1.253406773 |
| Q8N653 | LZTR1 | Leucine-zipper-like transcriptional regulator 1 | 1.253380617 |
| Q7Z7N9 | TMEM179B | Transmembrane protein 179B | 1.253120454 |
| P36507 | MAP2K2 | Dual specificity mitogen-activated protein kinase kinase 2 | 1.252605327 |
| Q13268 | DHRS2 | Dehydrogenase/reductase SDR family member 2, mitochondrial | 1.251813455 |
| P53999 | SUB1 | Activated RNA polymerase II transcriptional coactivator p15 | 1.248839311 |
| Q9H1E3 | NUCKS1 | Nuclear ubiquitous casein and cyclin-dependent kinase substrate 1 | 1.248676421 |
| Q9H788 | SH2D4A | SH2 domain-containing protein 4A | 1.247815744 |
| Q8IWC1 | MAP7D3 | MAP7 domain-containing protein 3 | 1.247372034 |
| P43115 | PTGER3 | Prostaglandin E2 receptor EP3 subtype | 1.246879059 |
| Q6ZN18 | AEBP2 | Zinc finger protein AEBP2 | 1.24648453 |
| Q8NI35 | PATJ | InaD-like protein | 1.24623144 |
| Q9Y4F5 | CEP170B | Centrosomal protein of 170 kDa protein B | 1.245492435 |

| | | | |
|---|---|---|---|
| Q8N128 | FAM177A1 | Protein FAM177A1 | 1.245199779 |
| Q7L4I2 | RSRC2 | Arginine/serine-rich coiled-coil protein 2 | 1.244248862 |
| P07205 | PGK2 | Phosphoglycerate kinase 2 | 1.243494777 |
| Q13573 | SNW1 | SNW domain-containing protein 1 | 1.242602105 |
| Q15678 | PTPN14 | Tyrosine-protein phosphatase non-receptor type 14 | 1.241718451 |
| Q9Y316 | MEMO1 | Protein MEMO1 | 1.241687677 |
| Q15398 | DLGAP5 | Disks large-associated protein 5 | 1.241359914 |
| P29373 | CRABP2 | Cellular retinoic acid-binding protein 2 | 1.239320246 |
| Q9BW85 | YJU2 | YJU2 splicing factor homolog | 1.238269443 |
| P48047 | ATP5O | ATP synthase subunit O, mitochondrial | 1.23680797 |
| P46937 | YAP1 | Transcriptional coactivator YAP1 | 1.236755164 |
| Q8TF05 | PPP4R1 | Serine/threonine-protein phosphatase 4 regulatory subunit 1 | 1.235827235 |
| P14174 | MIF | Macrophage migration inhibitory factor | 1.235406376 |
| Q86YP4 | GATAD2A | Transcriptional repressor p66-alpha | 1.234582282 |
| Q86V81 | ALYREF | THO complex subunit 4 | 1.234053994 |
| P23528 | CFL1 | Cofilin-1 | 1.233962302 |
| Q9NQ88 | TIGAR | Fructose-2,6-bisphosphatase TIGAR | 1.233681399 |
| P62249 | RPS16 | 40S ribosomal protein S16 | 1.233594379 |
| Q8N257 | HIST3H2BB | Histone H2B type 3-B | 1.23305659 |
| Q9BSD7 | NTPCR | Cancer-related nucleoside-triphosphatase | 1.232872203 |
| Q00059 | TFAM | Transcription factor A, mitochondrial | 1.232248125 |
| Q16775 | HAGH | Hydroxyacylglutathione hydrolase, mitochondrial | 1.231666132 |
| Q9UKY7 | CDV3 | Protein CDV3 homolog | 1.23165581 |
| Q15785 | TOMM34 | Mitochondrial import receptor subunit TOM34 | 1.230957984 |
| Q5JU85 | IQSEC2 | IQ motif and SEC7 domain-containing protein 2 | 1.229717616 |
| Q9NUQ6 | SPATS2L | SPATS2-like protein | 1.229699318 |
| P13693 | TPT1 | Translationally-controlled tumor protein | 1.228899859 |
| Q9BW83 | IFT27 | Intraflagellar transport protein 27 homolog | 1.227714854 |
| Q9NSI2 | FAM207A | Protein FAM207A | 1.226779957 |
| O75347 | TBCA | Tubulin-specific chaperone A | 1.225323104 |
| Q9GZU8 | FAM192A | Protein FAM192A | 1.225214573 |
| Q8WXX5 | DNAJC9 | DnaJ homolog subfamily C member 9 | 1.224468646 |
| Q14241 | ELOA | Elongin-A | 1.22408387 |
| Q96RU3 | FNBP1 | Formin-binding protein 1 | 1.223685746 |
| Q92665 | MRPS31 | 28S ribosomal protein S31, mitochondrial | 1.22356098 |
| Q7Z7A3 | CTU1 | Cytoplasmic tRNA 2-thiolation protein 1 | 1.222682175 |
| Q5U5X0 | LYRM7 | Complex III assembly factor LYRM7 | 1.222460414 |
| Q86SX3 | TEDC1 | Tubulin epsilon and delta complex protein 1 | 1.222402928 |
| Q9Y605 | MRFAP1 | MORF4 family-associated protein 1 | 1.222054027 |
| P67809 | YBX1 | Nuclease-sensitive element-binding protein 1 | 1.221098797 |
| Q15056 | EIF4H | Eukaryotic translation initiation factor 4H | 1.22035531 |
| P16104 | H2AFX | Histone H2AX | 1.220301231 |
| Q6P597 | KLC3 | Kinesin light chain 3 | 1.220071365 |
| O75438 | NDUFB1 | NADH dehydrogenase [ubiquinone] 1 beta subcomplex subunit 1 | 1.218323423 |

| | | | |
|---|---|---|---|
| O43869 | OR2T1 | Olfactory receptor 2T1 | 1.217257355 |
| Q92890 | UFD1 | Ubiquitin recognition factor in ER-associated degradation protein 1 | 1.217034001 |
| P49914 | MTHFS | 5-formyltetrahydrofolate cyclo-ligase | 1.21680543 |
| Q13310 | PABPC4 | Polyadenylate-binding protein 4 | 1.215944278 |
| P62979 | RPS27A | Ubiquitin-40S ribosomal protein S27a | 1.215538359 |
| P39019 | RPS19 | 40S ribosomal protein S19 | 1.215411145 |
| P11217 | PYGM | Glycogen phosphorylase, muscle form | 1.215091025 |
| Q5T0J7 | TEX35 | Testis-expressed protein 35 | 1.214670339 |
| P17844 | DDX5 | Probable ATP-dependent RNA helicase DDX5 | 1.214432238 |
| P62854 | RPS26 | 40S ribosomal protein S26 | 1.213852162 |
| P04350 | TUBB4A | Tubulin beta-4A chain | 1.21345528 |
| P52739 | ZNF131 | Zinc finger protein 131 | 1.213255521 |
| P0DP23 | CALM1 | Calmodulin-1 | 1.212858704 |
| O95858 | TSPAN15 | Tetraspanin-15 | 1.212515434 |
| P49321 | NASP | Nuclear autoantigenic sperm protein | 1.212290647 |
| Q92804 | TAF15 | TATA-binding protein-associated factor 2N | 1.212153376 |
| Q9UEW8 | STK39 | STE20/SPS1-related proline-alanine-rich protein kinase | 1.211975842 |
| P37108 | SRP14 | Signal recognition particle 14 kDa protein | 1.211563615 |
| P11387 | TOP1 | DNA topoisomerase 1 | 1.210311639 |
| Q8NC51 | SERBP1 | Plasminogen activator inhibitor 1 RNA-binding protein | 1.210131801 |
| Q9NQ29 | LUC7L | Putative RNA-binding protein Luc7-like 1 | 1.209813614 |
| Q13185 | CBX3 | Chromobox protein homolog 3 | 1.209017436 |
| Q9UHA3 | RSL24D1 | Probable ribosome biogenesis protein RLP24 | 1.208673628 |
| P61586 | RHOA | Transforming protein RhoA | 1.208087113 |
| P63173 | RPL38 | 60S ribosomal protein L38 | 1.207837288 |
| P31689 | DNAJA1 | DnaJ homolog subfamily A member 1 | 1.207619163 |
| Q8NDD1 | C1orf131 | Uncharacterized protein C1orf131 | 1.207311341 |
| Q8NEZ3 | WDR19 | WD repeat-containing protein 19 | 1.206519718 |
| Q13228 | SELENBP1 | Methanethiol oxidase | 1.206085953 |
| Q15818 | NPTX1 | Neuronal pentraxin-1 | 1.205786198 |
| Q96C90 | PPP1R14B | Protein phosphatase 1 regulatory subunit 14B | 1.204824062 |
| Q8NEY1 | NAV1 | Neuron navigator 1 | 1.204636013 |
| Q96F63 | CCDC97 | Coiled-coil domain-containing protein 97 | 1.20449852 |
| O43252 | PAPSS1 | Bifunctional 3'-phosphoadenosine 5'-phosphosulfate synthase 1 | 1.204028572 |
| Q3T8J9 | GON4L | GON-4-like protein | 1.203576735 |
| Q9H5V9 | CXorf56 | UPF0428 protein CXorf56 | 1.203145848 |
| O95817 | BAG3 | BAG family molecular chaperone regulator 3 | 1.203083931 |
| Q16626 | MEA1 | Male-enhanced antigen 1 | 1.202481142 |
| Q9Y3B7 | MRPL11 | 39S ribosomal protein L11, mitochondrial | 1.202303557 |
| P39687 | ANP32A | Acidic leucine-rich nuclear phosphoprotein 32 family member A | 1.202219384 |
| Q9Y4P1 | ATG4B | Cysteine protease ATG4B | 1.202163735 |
| P02545 | LMNA | Prelamin-A/C | 1.201932112 |
| O14979 | HNRNPDL | Heterogeneous nuclear ribonucleoprotein D-like | 1.201834809 |

| | | | |
|---|---|---|---|
| P35269 | GTF2F1 | General transcription factor IIF subunit 1 | 1.201700967 |
| Q9NRG1 | PRTFDC1 | Phosphoribosyltransferase domain-containing protein 1 | 1.201408167 |
| P62829 | RPL23 | 60S ribosomal protein L23 | 1.201268576 |
| Q13885 | TUBB2A | Tubulin beta-2A chain | 1.200466376 |
| Q66PJ3 | ARL6IP4 | ADP-ribosylation factor-like protein 6-interacting protein 4 | 1.200172608 |
| Q3ZAQ7 | VMA21 | Vacuolar ATPase assembly integral membrane protein VMA21 | 0.832782621 |
| Q9BUM1 | G6PC3 | Glucose-6-phosphatase 3 | 0.832725967 |
| O94923 | GLCE | D-glucuronyl C5-epimerase | 0.832186111 |
| Q7Z5Q5 | POLN | DNA polymerase nu | 0.831617641 |
| P20742 | PZP | Pregnancy zone protein | 0.83152963 |
| Q96RD7 | PANX1 | Pannexin-1 | 0.831370269 |
| Q8TE02 | ELP5 | Elongator complex protein 5 | 0.831121022 |
| Q7L3T8 | PARS2 | Probable proline--tRNA ligase, mitochondrial | 0.8310823 |
| P46977 | STT3A | Dolichyl-diphosphooligosaccharide--protein glycosyltransferase subunit STT3A | 0.830559413 |
| P78540 | ARG2 | Arginase-2, mitochondrial | 0.830279359 |
| Q9H9S5 | FKRP | Fukutin-related protein | 0.830261536 |
| P00846 | MT-ATP6 | ATP synthase subunit a | 0.829722048 |
| Q9H3Z4 | DNAJC5 | DnaJ homolog subfamily C member 5 | 0.829547542 |
| Q96E22 | NUS1 | Dehydrodolichyl diphosphate synthase complex subunit NUS1 | 0.829483844 |
| O43915 | VEGFD | Vascular endothelial growth factor D | 0.828244645 |
| Q9BRT2 | UQCC2 | Ubiquinol-cytochrome-c reductase complex assembly factor 2 | 0.827465918 |
| Q9H5Q4 | TFB2M | Dimethyladenosine transferase 2, mitochondrial | 0.827235437 |
| Q8NFV4 | ABHD11 | Protein ABHD11 | 0.826893135 |
| Q86W28 | NLRP8 | NACHT, LRR and PYD domains-containing protein 8 | 0.826277232 |
| Q9P0S3 | ORMDL1 | ORM1-like protein 1 | 0.826062828 |
| Q14118 | DAG1 | Dystroglycan | 0.825977351 |
| Q9NXN4 | GDAP2 | Ganglioside-induced differentiation-associated protein 2 | 0.825404298 |
| Q5BJH2 | TMEM128 | Transmembrane protein 128 | 0.824934367 |
| Q92609 | TBC1D5 | TBC1 domain family member 5 | 0.823697208 |
| Q5BJF2 | TMEM97 | Sigma intracellular receptor 2 | 0.823311651 |
| P43003 | SLC1A3 | Excitatory amino acid transporter 1 | 0.823250869 |
| P56282 | POLE2 | DNA polymerase epsilon subunit 2 | 0.823130724 |
| Q8WV93 | AFG1L | AFG1-like ATPase | 0.823080349 |
| P09493 | TPM1 | Tropomyosin alpha-1 chain | 0.823067091 |
| Q15051 | IQCB1 | IQ calmodulin-binding motif-containing protein 1 | 0.82273043 |
| Q9H553 | ALG2 | Alpha-1,3/1,6-mannosyltransferase ALG2 | 0.821977555 |
| Q15904 | ATP6AP1 | V-type proton ATPase subunit S1 | 0.821957119 |
| Q8WV99 | ZFAND2B | AN1-type zinc finger protein 2B | 0.821795463 |
| O00767 | SCD | Acyl-CoA desaturase | 0.818973335 |
| Q14728 | MFSD10 | Major facilitator superfamily domain-containing protein 10 | 0.818263868 |

| | | | |
|---|---|---|---|
| O43657 | TSPAN6 | Tetraspanin-6 | 0.817291474 |
| P42262 | GRIA2 | Glutamate receptor 2 | 0.817046231 |
| Q9UIS9 | MBD1 | Methyl-CpG-binding domain protein 1 | 0.816872887 |
| Q9UFH2 | DNAH17 | Dynein heavy chain 17, axonemal | 0.815786939 |
| Q96GJ1 | TRMT2B | tRNA (uracil(54)-C(5))-methyltransferase homolog | 0.814785738 |
| Q07075 | ENPEP | Glutamyl aminopeptidase | 0.814424154 |
| Q9H9H4 | VPS37B | Vacuolar protein sorting-associated protein 37B | 0.813843603 |
| P61803 | DAD1 | Dolichyl-diphosphooligosaccharide--protein glycosyltransferase subunit DAD1 | 0.813294932 |
| Q7Z3Z4 | PIWIL4 | Piwi-like protein 4 | 0.812560233 |
| Q8NHP8 | PLBD2 | Putative phospholipase B-like 2 | 0.812152256 |
| Q8N2K0 | ABHD12 | Monoacylglycerol lipase ABHD12 | 0.812047838 |
| P07996 | THBS1 | Thrombospondin-1 | 0.811844003 |
| O75449 | KATNA1 | Katanin p60 ATPase-containing subunit A1 | 0.811064652 |
| Q12770 | SCAP | Sterol regulatory element-binding protein cleavage-activating protein | 0.811015248 |
| Q9P2W1 | PSMC3IP | Homologous-pairing protein 2 homolog | 0.810824422 |
| O00584 | RNASET2 | Ribonuclease T2 | 0.810520464 |
| Q9NRM1 | ENAM | Enamelin | 0.810374692 |
| P56134 | ATP5J2 | ATP synthase subunit f, mitochondrial | 0.809812941 |
| Q69YH5 | CDCA2 | Cell division cycle-associated protein 2 | 0.809240805 |
| Q15118 | PDK1 | [Pyruvate dehydrogenase (acetyl-transferring)] kinase isozyme 1, mitochondrial | 0.807523833 |
| P18085 | ARF4 | ADP-ribosylation factor 4 | 0.807086646 |
| Q5JSL3 | DOCK11 | Dedicator of cytokinesis protein 11 | 0.805761678 |
| Q14108 | SCARB2 | Lysosome membrane protein 2 | 0.804760212 |
| Q9H6K4 | OPA3 | Optic atrophy 3 protein | 0.804190461 |
| Q9Y221 | NIP7 | 60S ribosome subunit biogenesis protein NIP7 homolog | 0.803863387 |
| Q9H2D1 | SLC25A32 | Mitochondrial folate transporter/carrier | 0.803467367 |
| Q86TJ2 | TADA2B | Transcriptional adapter 2-beta | 0.803350941 |
| P12645 | BMP3 | Bone morphogenetic protein 3 | 0.802466945 |
| Q8WTV0 | SCARB1 | Scavenger receptor class B member 1 | 0.801482955 |
| Q9Y6K0 | CEPT1 | Choline/ethanolaminephosphotransferase 1 | 0.801353643 |
| P12235 | SLC25A4 | ADP/ATP translocase 1 | 0.79831037 |
| Q16563 | SYPL1 | Synaptophysin-like protein 1 | 0.798075113 |
| P13645 | KRT10 | Keratin, type I cytoskeletal 10 | 0.797779412 |
| Q9Y3E0 | GOLT1B | Vesicle transport protein GOT1B | 0.797708279 |
| P02549 | SPTA1 | Spectrin alpha chain, erythrocytic 1 | 0.797692713 |
| O43861 | ATP9B | Probable phospholipid-transporting ATPase IIB | 0.797331906 |
| Q8IZ40 | RCOR2 | REST corepressor 2 | 0.797242036 |
| Q99640 | PKMYT1 | Membrane-associated tyrosine- and threonine-specific cdc2-inhibitory kinase | 0.797195019 |
| Q8N3A8 | PARP8 | Poly [ADP-ribose] polymerase 8 | 0.797117318 |
| Q9Y5B6 | PAXBP1 | PAX3- and PAX7-binding protein 1 | 0.795897418 |
| Q8IV08 | PLD3 | Phospholipase D3 | 0.793494531 |
| P32926 | DSG3 | Desmoglein-3 | 0.792615884 |

| | | | |
|---|---|---|---|
| Q9Y3C1 | NOP16 | Nucleolar protein 16 | 0.78827528 |
| Q9H3U5 | MFSD1 | Major facilitator superfamily domain-containing protein 1 | 0.787879985 |
| P18440 | NAT1 | Arylamine N-acetyltransferase 1 | 0.787215966 |
| Q0P6H9 | TMEM62 | Transmembrane protein 62 | 0.787004516 |
| Q6IE37 | OVOS1 | Ovostatin homolog 1 | 0.785739729 |
| P14854 | COX6B1 | Cytochrome c oxidase subunit 6B1 | 0.78573252 |
| Q7LGC8 | CHST3 | Carbohydrate sulfotransferase 3 | 0.785139337 |
| Q9UIU6 | SIX4 | Homeobox protein SIX4 | 0.785034589 |
| Q8NF50 | DOCK8 | Dedicator of cytokinesis protein 8 | 0.783660065 |
| P35527 | KRT9 | Keratin, type I cytoskeletal 9 | 0.777007683 |
| Q9NVV0 | TMEM38B | Trimeric intracellular cation channel type B | 0.776968774 |
| Q8WV60 | PTCD2 | Pentatricopeptide repeat-containing protein 2, mitochondrial | 0.774537651 |
| P55160 | NCKAP1L | Nck-associated protein 1-like | 0.769948494 |
| A6NJI9 | LRRC72 | Leucine-rich repeat-containing protein 72 | 0.769321389 |
| Q96E29 | MTERF3 | Transcription termination factor 3, mitochondrial | 0.7684804 |
| Q14703 | MBTPS1 | Membrane-bound transcription factor site-1 protease | 0.764019643 |
| Q8WV22 | NSMCE1 | Non-structural maintenance of chromosomes element 1 homolog | 0.762571266 |
| P02533 | KRT14 | Keratin, type I cytoskeletal 14 | 0.762261094 |
| Q99683 | MAP3K5 | Mitogen-activated protein kinase kinase kinase 5 | 0.760636019 |
| Q8N2E2 | VWDE | von Willebrand factor D and EGF domain-containing protein | 0.75919479 |
| Q658P3 | STEAP3 | Metalloreductase STEAP3 | 0.754495043 |
| Q9NR77 | PXMP2 | Peroxisomal membrane protein 2 | 0.75430007 |
| Q9H061 | TMEM126A | Transmembrane protein 126A | 0.753290141 |
| A2RU67 | FAM234B | Protein FAM234B | 0.751353893 |
| Q9Y5L4 | TIMM13 | Mitochondrial import inner membrane translocase subunit Tim13 | 0.746474784 |
| O75385 | ULK1 | Serine/threonine-protein kinase ULK1 | 0.742344796 |
| Q9BY50 | SEC11C | Signal peptidase complex catalytic subunit SEC11C | 0.740168839 |
| P02042 | HBD | Hemoglobin subunit delta | 0.738533281 |
| Q14534 | SQLE | Squalene monooxygenase | 0.738456958 |
| P30536 | TSPO | Translocator protein | 0.734624502 |
| Q9P2K6 | KLHL42 | Kelch-like protein 42 | 0.733512268 |
| P02768 | ALB | Serum albumin | 0.731421853 |
| Q4W5G0 | TIGD2 | Tigger transposable element-derived protein 2 | 0.729096849 |
| Q6UX72 | B3GNT9 | UDP-GlcNAc:betaGal beta-1,3-N-acetylglucosaminyltransferase 9 | 0.716845933 |
| Q96BM9 | ARL8A | ADP-ribosylation factor-like protein 8A | 0.715510626 |
| P57057 | SLC37A1 | Glucose-6-phosphate exchanger SLC37A1 | 0.713464264 |
| Q9UJX2 | CDC23 | Cell division cycle protein 23 homolog | 0.710770783 |
| O15050 | TRANK1 | TPR and ankyrin repeat-containing protein 1 | 0.708247483 |
| Q02413 | DSG1 | Desmoglein-1 | 0.707750707 |
| P28330 | ACADL | Long-chain specific acyl-CoA dehydrogenase, mitochondrial | 0.707497167 |

| | | | |
|---|---|---|---|
| P80365 | HSD11B2 | Corticosteroid 11-beta-dehydrogenase isozyme 2 | 0.700629987 |
| Q9P003 | CNIH4 | Protein cornichon homolog 4 | 0.696978494 |
| Q92564 | DCUN1D4 | DCN1-like protein 4 | 0.691796699 |
| Q9NX00 | TMEM160 | Transmembrane protein 160 | 0.685147795 |
| Q96EU6 | RRP36 | Ribosomal RNA processing protein 36 homolog | 0.680711277 |
| P78332 | RBM6 | RNA-binding protein 6 | 0.656667186 |
| P04264 | KRT1 | Keratin, type II cytoskeletal 1 | 0.656660409 |
| P21860 | ERBB3 | Receptor tyrosine-protein kinase erbB-3 | 0.643736939 |
| P08779 | KRT16 | Keratin, type I cytoskeletal 16 | 0.633121401 |
| Q5HY64 | FAM47C | Putative protein FAM47C | 0.625502861 |
| P13647 | KRT5 | Keratin, type II cytoskeletal 5 | 0.611197075 |
| P51786 | ZNF157 | Zinc finger protein 157 | 0.586087661 |
| P48668 | KRT6C | Keratin, type II cytoskeletal 6C | 0.56390028 |
| Q92858 | ATOH1 | Protein atonal homolog 1 | 0.561748688 |
| Q9UL62 | TRPC5 | Short transient receptor potential channel 5 | 0.560397651 |
| P78369 | CLDN10 | Claudin-10 | 0.553699125 |
| Q9H8M5 | CNNM2 | Metal transporter CNNM2 | 0.537252179 |
| O95674 | CDS2 | Phosphatidate cytidylyltransferase 2 | 0.490415498 |
